# Magnetic resonance imaging findings in COVID-19-related anosmia

**DOI:** 10.55730/1300-0144.5490

**Published:** 2021-10-05

**Authors:** Hüseyin ÇETİN, Ayşe Şule ATEŞ, Ogün TAYDAŞ, Bahri ELMAS, Ertuğrul GÜÇLÜ

**Affiliations:** 1 Department of Radiology, Faculty of Medicine, Ankara Yıldırım Beyazıt University, Ankara, Turkey; 2 Department of Chest Diseases, Sakarya University Training and Research Hospital, Sakarya, Turkey; 3 Department of Family Medicine, University of Health Sciences, Haydarpaşa Numune Training and Research Hospital, İstanbul, Turkey; 4 Department of Pediatrics, Faculty of Medicine, Sakarya University, Sakarya, Turkey; 5 Department of Infectious Diseases and Clinical Microbiology, Faculty of Medicine, Sakarya University, Sakarya, Turkey

**Keywords:** COVID-19, anosmia, magnetic resonance imaging

## Abstract

**Background/aim:**

The coronavirus disease 2019 (COVID-19) mostly manifests with fever, shortness of breath, and cough, has also been found to cause some neurological symptoms, such as anosmia and ageusia. The aim of the study was to present the magnetic resonance imaging (MRI) findings of patients with anosmia-hyposmia symptoms and to discuss potential mechanisms in light of these findings.

**Materials and methods:**

Of the 2412 patients diagnosed with COVID-19-related pneumonia (RT-PCR at least once + clinically confirmed) between March and December 2020, 15 patients underwent olfactory MRI to investigate the cause of ongoing anosmia/hyposmia symptoms were included in the study.

**Results:**

Eleven (73.3%) patients were female and four (26.7%) were male. A total of eight patients (53.3%) showed thickening in the olfactory cleft region, where the olfactory epithelium is located. In nine patients (60%), enhancement was observed in the olfactory cleft region. Diffusion-weighted imaging showed restricted diffusion in three patients (20%) (corpus callosum splenium in one patient, thalamus mediodorsal nucleus in one patient, and mesencephalon in one patient).

**Conclusion:**

This study revealed that there is a relationship between anosmia and MRI findings. Larger studies can enlighten the pathophysiological mechanism and shed light on both diagnosis and new treatments.

## 1. Introduction

The coronavirus disease 2019 (COVID-19), which started spreading in December 2019, rapidly became a pandemic. The disease, which mostly manifests with fever, shortness of breath, and cough, has also been found to cause some neurological symptoms, such as anosmia, ageusia, headache, and confusion [1].

Although it varies in various publications, a significant portion (5%–68%) of COVID-19-positive patients have been shown to have symptoms related to the sense of smell and taste [2–4]. In fact, a study showed that almost all COVID-19 patients had loss of sense of smell at a stage close to the end of the acute recovery period, albeit at different levels [5]. Moreover, while this loss was temporary in many patients, in some patients, it was irreversible [6]. It has been observed that olfactory dysfunction may occur before general symptoms, as well as during the course of the disease or following other symptoms [7].

Studies showing that the viral load was more intense in the nasal mucosa than the throat suggested that the nasal epithelium might be an important location for the infection to start and for the virus to proliferate and spread [8]. Similar to severe acute respiratory syndrome-associated coronavirus (SARS-CoV), the current virus has also been shown to utilize angiotensin-converting enzyme-2 (ACE2) on the cell surface as a receptor to enter the cell [9]. Immunohistochemical studies have revealed that these enzymes are found in the nasal and bronchial epithelium [10]. This information suggests that the effects on the nasal mucosa and the olfactory epithelium in the superior nasal meatus of SARS-CoV2 may be the mechanisms causing smell-related symptoms, but this still does not explain why in COVID-19, the smell-taste disorder is much more frequent than SARS-CoV infections that use a similar mechanism to enter the cell or other infections, such as influenza that cause smell and taste disorder through upper respiratory tract infections; thus, the true mechanism of the new virus has not yet been fully elucidated [11].

In some published articles, COVID-19 has been shown to involve the central nervous system, cause posterior reversible encephalopathy syndrome-like findings in the brain, or lead to the development of acute necrotizing encephalopathy [12]. In addition, in a published case report, a patient with anosmia symptom presented with signal changes on brain MRI images [13]. Moreover, studies have reported that SARS-CoV infections cause neuronal death, especially the death of the brain stem [14]. In light of this information, another hypothesis is that in COVID-19, smell and taste disorders may occur due to central involvement.

Imaging methods play an important role in clarifying these mechanisms and better understanding COVID-19 infections. The aim of the study was to present the MRI findings of COVID-19 patients with anosmia-hyposmia symptoms and to discuss potential mechanisms in light of these findings.

## 2. Materials and methods

For this retrospective study, approval was obtained from the ethical committee of our institution.

### 2.1. Patient selection

Of the 2412 patients diagnosed with COVID-19 pneumonia (confirmed by a minimum of one positive RT-PCR test + clinical findings) between March and December 2020 who underwent olfactory MRI to investigate the cause of the ongoing anosmia/hyposmia symptoms were included in the study. The sino-nasal outcome test -22 (SNOT-22) questionnaire, which was validated in the Turkish population, was used for smell and taste assessment [15].

### 2.2. MRI examinations

MRI was performed with a 1.5-T system (Signa Voyager; GE Healthcare, Milwaukee, WI, USA). The image acquisition protocol included (1) 3 mm-thick T2-weighted images without interslice gap in the coronal plane covering the whole brain using the fast spin-echo sequence (TR 2,430 ms; TW = 107 ms); (2) 5 mm-thick diffusion-weighted images with a 1-mm interslice gap in the axial plane covering the whole brain (TR 6400, TE = 98 ms, b values 0 and 1,000 s/mm2), and (3) 0.6 mm-thick 3D FIESTA-C images (TR: 5.4, TE: 2.1 ms, FOV 150 mm × 100 mm, 146 axial slices), and 0.6 mm-thick precontrast and postcontrast 3D T1-weighted BRAVO images (TR: 1900, TE: 2.55 ms, FOV 150 mm × 100 mm, 146 axial slices).

### 2.3. Image analysis

All imaging findings were evaluated simultaneously, including complete nasal obstruction, superior orbital meatus obstruction, and sinonasal mucosa, excessive enhancement in postcontrast images, pathological signal change in T2-weighted images in the brain, and diffusion restriction in the brain. Orbital cleft thickness was evaluated on coronal T2-weighted images. The evaluation of the images was independently undertaken by two radiologists with the prediagnosis of COVID-19-related pneumonia. If their initial opinions differed, a consensus was reached.

### 2.4. Statistical analysis

MedCalc (ver. 12, Ostend, Belgium) was used for statistical analysis. The descriptive statistics were given as median (minimum–maximum) and mean ± standard deviation. Categorical variables were expressed as frequencies and percentages.

## 3. Results

**Figure 1:**
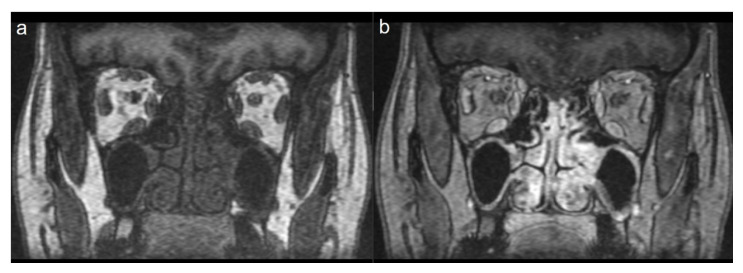
Coronal pre- (a) and postcontrast (b) T1-weighted image showing thickening and enhancement in the bilateral olfactory cleft and mucosa.

**Figure 2:**
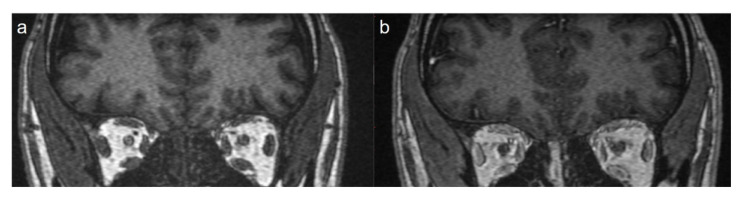
Coronal pre- (a) and postcontrast (b) T1-weighted image showing thickening and enhancement in the right olfactory cleft and mucosa.

**Figure 3:**
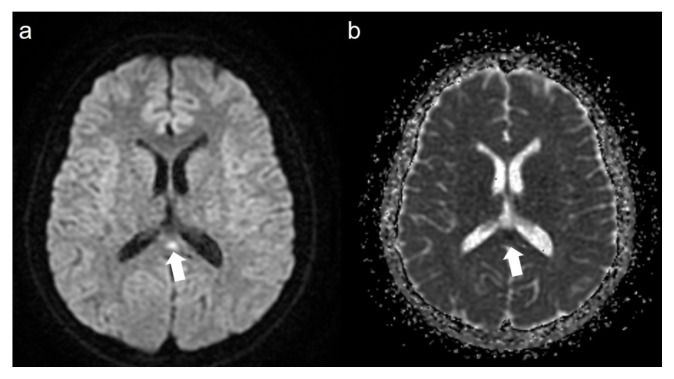
Axial diffusion-weighted image (a) (b = 1000) and the apparent diffusion coefficient (ADC) map (b) showing a diffusion-restricted lesion in the splenium of the corpus callosum (arrow).

**Figure 4:**
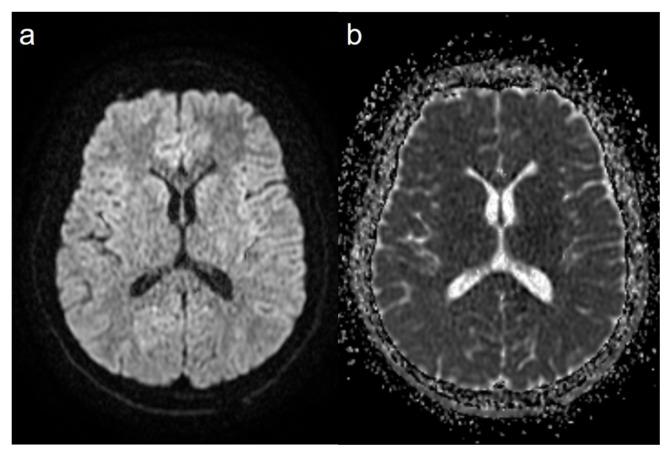
The control images of the same patient, axial diffusion-weighted image (a) (b = 1000) and the apparent diffusion coefficient (ADC) map (b), shows that the lesions disappeared.

A total of 15 patients were included in the study. Eleven (73.3%) of the patients were female and 4 (26.7%) were male. The mean age of the patients was 25.1 ± 11.4 years. When the pneumonia findings were evaluated, disease severity was mild in 13 patients (86.6%) and moderate in two patients (13.4%). The average length of hospital stay was 3 ± 1.9 days. A total of eight patients (53.3%) showed thickening in the olfactory cleft region, where the olfactory epithelium is located. In five of these patients (62.5%) thickening was bilateral (Figures 1a and 1b), while in three patients, thickening was unilateral (37.5%) (Figures 2a and 2b). In nine patients (60%), enhancement was observed in the olfactory cleft region. Enhancement was bilateral in five patients (55.5%), and unilateral in four patients (44.5%). Diffusion-weighted imaging was restricted in three patients (20%) (in the corpus callosum splenium, thalamus mediodorsal nucleus and mesencephalon in one patient each). In these three patients, T2 signal was also increased in areas with restricted diffusion. However, no T2 signal changes were detected in the brain in other patients. In one of our patients, the smell disorder appeared as a symptom of mild encephalitis/encephalopathy with a reversible splenial lesion (MERS) (Figures 3a, 3b and 4a, 4b). None of the patients had nasal obstruction. While only one patient (6.6%) had a runny nose, five (33.3%) had postnasal drip and six (40%) had a sore throat. Anosmia was present in 10 patients (66.6%) and hyposmia in five patients (33.4%). The mean symptom duration of the patients was 11.4 ± 6.3 days. Complete improvement was achieved in 12 patients (80%), and partial in three patients (20%). The most common symptom related to the sense of taste was hypogeusia detected in seven patients (46.6%). Three patients (20%) had ageusia (Table).

## 4. Discussion

In approximately half of the patients (8/15), we observed unilateral (3/8) or bilateral (5/8) thickening in the olfactory cleft region and intense contrast enhancement in this area in nine patients. In addition, in three patients, we found restricted diffusion findings in the corpus callosum splenium, thalamus mediodorsal nucleus and mesencephalon in diffusion-weighted images. In five of the 15 patients, we did not find any pathological findings on MRI.

Olfactory system disorder is a very common condition in COVID-19 infection. Although many agents of upper respiratory tract infections (parainfluenza, rhinovirus, influenza, and coronavirus) are known to cause loss of smell due to olfactory epithelial involvement, this symptom has been reported to be more common in COVID-19 patients [4,16]. In addition, unlike upper respiratory infections, COVID-19 also rarely shows upper respiratory symptoms [17]. As in our study, it was very interesting to observe anosmia without nasal obstruction in COVID-19 patients. None of our patients had nasal obstruction clinically or radiologically. In addition, although some of the patients with radiological olfactory cleft involvement had narrowing of this region, only a small portion of olfactory cleft obstruction was observed (2/15). Although the loss of sense of smell due to viral factors is mostly recovered in the early period, it is known to last up to two years in some patients [18]. In a recent study of 23 patients by Kandemirli et al., diffusely increased signal intensity, scattered hyperintense foci or microhemorrhages were observed in 91.3% of the patients [19]. In a recent study by Chetrit et al., bilateral obstruction of the olfactory clefts was detected in 47% of COVID-19 patients with sudden-onset total loss of smell [20]. In all of the current study patients, inflammatory thickening and increased T2 signal were observed in the nasal mucosa at the olfactory cleft. In a study by Strauss et al., the normalized olfactory bulb T2 FLAIR (fluid-Attenuated inversion recovery) signal intensity value was found to be higher in patients with anosmia. However, in that study, only 4 of 12 patients had intraneural T2 signal hyperintensity on 3D T2 FLAIR [21].

There are very few imaging studies on the loss of sense of smell developing during or after upper respiratory tract infections. In a study by Muller et al. evaluating patients with olfactory dysfunction caused by upper respiratory infections, it was observed that the volume of the olfactory bulbus was greater in patients with hyposmia than in those with complete anosmia [22]. Yao et al. also showed that the olfactory bulb volume decreased in patients with postviral smell loss [23]. Chung et al. investigated whether there was atrophy by evaluating the olfactory bulb volume in patients, and whether there was neuropathy by evaluating the olfactory bulb signal intensity in FLAIR MRI images. In postviral cases, there were signs of atrophy and neuropathy in patients with both anosmia/hyposmia and normosmia, and there was no significant difference between these two groups [24]. However, in that study, the number of postviral patients was very low.

The cell surfaces of proteins/receptors to which viruses attach and the mechanisms that allow viruses to enter these cells are the main factors that determine the involvement and spread regions of infections. The viral load in nasal swabs being shown to be higher than the samples taken from the throat indicates that the nasal mucosa can be very effective in the introduction of the SARS-CoV2 virus and its spread from person to person [25]. Subsequent studies have shown that the spike protein binds to the ACE2 receptor on the cell surface and that S protein and ACE2 affinity is the major factor in virus proliferation and disease progression [9]. Infection of olfactory sustentacular cells in the olfactory cleft mucosa that does not allow the scent particles to reach the olfactory nerve may be one of the mechanisms preventing smell. However, in a study of mice with another human coronavirus, virus antigens were observed three days after virus infection in the olfactory bulb, and seven days later, the olfactory symptoms were found not be limited to mucosal involvement. This is supported by our results revealing thickening and enhancement in the olfactory cleft although these findings were not present in most of the remaining mucosal tissue and sinuses. In addition, intense enhancement without significant mucosal thickening in the olfactory epithelium was seen in one patient, which could indicate that the effect is not only at the mucosal level. Politi et al. recently published the report of a case with anosmia, in which there were hyperintense signal changes in FLAIR MRI images in the gyrus rectus that disappeared after the patient’s symptoms regressed, suggesting that COVID-related odor disorders may also be associated with central involvement [13].

In one of our patients, there was hyperintensity and restricted diffusion in the T2-weighted image in the corpus callosum splenium without abnormal thickening or enhancement in the nasal olfactory cleft. This patient had no neurological symptom other than headache accompanying hyposmia. In addition, the patient had no respiratory symptoms. The control images obtained after the patient’s odor symptom disappeared (on the 17th day) no longer showed hyperintensity and restricted diffusion on the T2-weighted image. Based on these findings, the patient was diagnosed with mild encephalitis/encephalopathy with a reversible splenial lesion (MERS). Transient lesions of the corpus callosum can occur for many reasons, such as multiple sclerosis, trauma, drug use, and neoplasms, as well as viral etiology. In MERS, usually presenting with neurological symptoms, such as loss of consciousness, headache, and speech disorders after prodromal symptoms (e.g., fever, vomiting and diarrhea), the complete disappearance of neurological and MRI findings is typical [26]. It is interesting to note that in our patient, the symptoms were limited to headache and olfactory findings. Since the corpus callosum is a structure consisting of fibers that provide interhemispheric connection, smell-related symptoms rarely expected in these lesions. However, in a study of patients with corpus callosum agenesis, some patients were shown to have smell and taste impairment [27]. In addition, in an influenza-associated case of MERS reported by Takatsu et al., a significant decrease was observed in the sense of smell, as in our case, in addition to the change of consciousness [28]. To the best of our knowledge, the current report describes the first pediatric MERS case associated with COVID-19.

The thalamus is a structure associated with many functions, such as sensory perception, attention, sleep, and cognitive and behavioral memory. Although olfactory sensory neurons are not directly related to the thalamus, the mediodorsal thalamic nucleus is known to have afferent and efferent connections with both primary and secondary olfactory domains [29]. However, the effect of the thalamus on the olfactory system is not yet fully understood [30]. In our study, diffusion restriction in the thalamus mediodorsal nucleus was observed in one patient. Bilateral olfactory cleft involvement was also present in this patient. This patient did not present with additional neurological findings, and her olfactory deficit may have been due to mediodorsal nucleus involvement or olfactory cleft involvement. In a previous COVID-19 case with acute necrotizing hemorrhagic encephalopathy, bilateral thalamus involvement was also present [12]. Our patient had unilateral thalamic involvement, and there were no noteworthy imaging findings in other areas of the brain.

In the patient population in our study, it was observed that the ratios of young age and women were higher. Although different rates of age and sex are seen in previous studies conducted, COVID-19 patients with odor symptoms are mostly young, and this disease is more common in women according to a multicultural study conducted by Lechien et al. [7].

In one of our patients, we observed a focal restricted diffusion finding at the level of the superior cerebellar pedicle-mesencephalon junction. Although the superior cerebellar pedicle mainly contains fibers belonging to the cerebellothalamic tract, it is associated with the ventral nuclei of the thalamus, and there is no information related to the sense of smell. Therefore, in light of current information, we do not think that the restricted diffusion finding in this patient was directly related to the sense of smell. However, this finding is very important in terms of showing the central nervous system involvement of the disease. We consider that the deterioration in the sense of smell might be central in this case, in which we did not detect olfactory cleft involvement. In addition, since we do not know at which stage the neural involvement of the disease was at the time of acquiring the image, active inflammation in the areas related to the sense of smell may not have been displayed at the time when it exhibited restricted diffusion findings.

There were some limitations to our study. The first was the small number of patients included in the study, and the second was the retrospective nature of the research. More comprehensive and prospective studies should be planned on this subject.

Loss of sense of taste and smell has become increasingly important diagnostic findings for COVID-19. However, the subjective nature of this finding creates difficulties in explaining the pathophysiology. Our study revealed that there is a relationship between loss of sense of taste and smell and MRI findings. Furthermore, in one of our patients, the smell disorder appeared as a symptom of MERS, and to our knowledge, this is the first reported SARS-CoV2-related pediatric MERS case. Larger studies in the future can enlighten the pathophysiological mechanism and shed light on both diagnosis and new treatments.

## Figures and Tables

**Table:** Patient data.

Age/Sex	Diffusion-weighted imaging features	Olfactory cleft thickening	Enhancement in the olfactory cleft	Sense of smell	Sense of taste
30/F	-	Bilateral	Bilateral	Anosmia	Ageusia
29/F	-	Bilateral	Bilateral	Hyposmia	Hypogeusia
37/M	-	Unilateral	Unilateral	Anosmia	Normal
27/F	Mediodorsal nucleus	Bilateral	Bilateral	Anosmia	Normal
54/F	-	-	-	Hyposmia	Normal
27/F	-	-	-	Anosmia	Ageusia
27/F	-	Bilateral	Bilateral	Anosmia	Normal
21/F	-	-	-	Hyposmia	Hypogeusia
36/F	-	Unilateral	Unilateral	Anosmia	Normal
17/F	-	-	-	Anosmia	Hypogeusia
17/M	-	Bilateral	Bilateral	Anosmia	Hypogeusia
15/F	-	-	-	Anosmia	Ageusia
11/F	-	Unilateral	Unilateral	Hyposmia	Hypogeusia
14/M	Splenium of the corpus callosum	-	-	Hyposmia	Hypogeusia
15/M	Mesencephalon	-	Unilateral	Anosmia	Hypogeusia
